# Benefits of Telerehabilitation for Patients With Severe Acquired Brain Injury: Promising Results From a Multicenter Randomized Controlled Trial Using Nonimmersive Virtual Reality

**DOI:** 10.2196/45458

**Published:** 2023-08-21

**Authors:** Rocco Salvatore Calabrò, Mirjam Bonanno, William Torregrossa, Luisa Cacciante, Antonio Celesti, Carmela Rifici, Paolo Tonin, Rosaria De Luca, Angelo Quartarone

**Affiliations:** 1 Istituto di Ricovero e Cura a Carattere Scientifico Centro Neurolesi Bonino Pulejo Messina Italy; 2 Laboratory of Healthcare Innovation Technology Istituto di Ricovero e Cura a Carattere Scientifico San Camillo Hospital Venezia Italy; 3 Department of Mathematics, Computer Science, Physics and Earth Science University of Messina Messina Italy; 4 Sant'Anna Institute Crotone Italy

**Keywords:** telerehabilitation, teleneuro-VRRS, virtual reality rehabilitation system, SABI, severe acquired brain injury, acquired brain injury, virtual reality, rehabilitation, neurorehabilitation, brain injury, neurology

## Abstract

**Background:**

In neurorehabilitation, the use of innovative technologies offers many opportunities to monitor and improve the health status of patients with severe acquired brain injury (SABI). Telerehabilitation allows for continuity of service through the entire rehabilitation cycle, including assessment, intervention, consultation, and education, affording early reintegration and positively enhancing the quality of life (QoL).

**Objective:**

The main purpose of this multicenter randomized controlled trial was to test the effectiveness of advanced training provided using a nonimmersive virtual reality rehabilitation system (ie, the VRRS HomeKit device) in improving functional outcomes in patients with SABI.

**Methods:**

In total, 40 patients with SABI and their 40 caregivers visiting 2 Italian rehabilitation centers were enrolled in the study protocol and randomized into 2 groups. Of the 40 patients, 20 (50%) underwent the experimental training using the VRRS HomeKit (teleneuro-VRRS group), whereas the other 20 (50%) were administered usual territorial rehabilitative treatments (UTRTs; control group). To investigate motor and neuropsychological functioning, patients with SABI were evaluated before (T0) and at the end of (T1) each training session by a multispecialist team through a complete clinical and psychometric battery: the Barthel Index (BI), the Tinetti Scale (TS), the Modified Ashworth Scale (MAS), the Montreal Cognitive Assessment (MoCa), the Frontal Assessment Battery (FAB), the Beck Depression Inventory II (BDI-II), the Short Form Health Survey 36 (SF-36), and the Psychological General Well-Being Index (PGWBI). In addition, the Caregiver Burden Inventory (CBI) was administered to each caregiver to investigate the emotional burden status.

**Results:**

The teleneuro-VRRS group achieved a statistically significant improvement in both general and motor outcomes, as well as psychological well-being and QoL, compared to the control group. In particular, the BI (*P*<.001), FAB (*P*<.001), and BDI-II (*P*<.001) were the outcome scales with the best improvement. The burden of caregivers also significantly improved in the teleneuro-VRRS group (CBI; *P*<.004). Between-group analysis showed statistical differences in the anxiety (effect size [ES]=0.85, *P*<.02) and self-control (ES=0.40, *P*<.03) subtests of the PGWBI and in the social role functioning (ES=0.85, *P*<.02) subtest of the SF-36, confirmed by quite medium and large ESs.

**Conclusions:**

Our results suggest that the VRRS is a suitable alternative tool or complementary tool or both to improve motor (level of functional independence) and cognitive (frontal/executive abilities) outcomes, reducing behavioral alterations (anxiety and depression symptoms) in patients with SABI, with a beneficial impact also on the caregivers’ burden distress management, mitigating distress and promoting positive aspects of caring.

**Trial Registration:**

ClinicalTrials.gov NCT03709875; https://classic.clinicaltrials.gov/ct2/show/NCT03709875

## Introduction

Severe acquired brain injury (SABI) is a leading cause of death and disability worldwide. SABI refers to damage to the brain, occurring from traumatic brain injury (TBI) and nontraumatic causes (eg, ischemic or hemorrhagic stroke or anoxia following cardiac arrest), with a coma state lasting at least 24 hours [1,2]. Functional recovery following SABI usually reaches its peak at around 6 months (usually 1 year for TBI) and begins to decline as far as 1 year after the injury [3], although sensory motor deficits and cognitive behavioral abnormalities can persist up to months or years. In particular, following SABI, patients usually present with moderate-to-severe hemiparesis, spasticity in the upper and lower limbs, and loss of trunk control that limit breathing and communication [4]. All these physical disabilities associated with cognitive impairment can cause significant handicaps and limit return to a normal and productive life. Cognitive dysfunctions involve attention processes, executive functions, memory abilities, reasoning and problem solving, and visual spatial cognition and are worsened by mood disorders as well as other behavioral problems, including impulsivity/aggressivity [5]. All these problems negatively affect not only the patient’s but also the caregiver’s quality of life (QoL), given that family members play a crucial role in the rehabilitation process, supporting the recovery of patients with SABI [6]. Thus, a specific neurorehabilitation pathway is necessary for these individuals with frailty and should also be planned postdischarge to ensure the continuity of care. Indeed, SABI motor and cognitive therapy should be as intensive and long lasting as possible to allow patients to achieve the best independence and QoL [7]. Notably, neurorehabilitation approaches may be classified into 2 main categories: conventional, using paper-and-pencil exercises (for cognitive tasks) and face-to-face physical interaction with the therapist (for motor tasks) [8,9], and advanced [10,11], using computer-assisted techniques, robotics, or virtual reality (VR) [12-14]. Both are based on the use of specific strategies to retrain or alleviate the cognitive [15] and motor [16] alterations in patients with SABI.

Generally, conventional techniques consist of manual/paper-and-pencil exercises with the physical presence of a therapist in a traditional setting, whereas VR exercises use a computer interface to train motor and cognitive functions in a gamelike setting [17,18].

However, the families of patients with SABI can face some concerns, such as limited access to appropriate health care services and outpatient clinics because of traveling costs and geographical barriers. This reduces the amount of care provided to patients [19] and consequently increases caregivers’ distress and burnout [20]. In this context, there is a growing literature on the use of telemedicine for remote assessment and clinical interventions, as well as in the promising field of telerehabilitation (TR). This latter can be applied as either a personalized [21-23] or a group [24] intervention to improve functional outcomes in SABI.

TR has been defined as “the delivery of rehabilitation services via information and communication technologies.” Recent evidence supports the feasibility and utility of a home-based system to effectively deliver TR, improve patients’ outcomes, screen for complications of the disease, and provide the patients with a means for at-home interaction with medical personnel [25].

In the past few years, it has been shown that VR technology could be useful to stimulate recovery following a stroke [26] and TBI [27]. VR training offers the advantage of simulating daily activities according to patients’ needs in order to motivate them by avoiding boredom and frustration [28,29], although it requires compliance by the patients. Additionally, the use of nonimmersive VR tools is more advantageous in being cheaper and easier to use than immersive VR systems.

However, there is still a lack of studies on TR and nonimmersive VR training for cognitive and motor outcomes in patients with SABI. To this end, our study could fill this gap in the neurorehabilitation field. In fact, the concomitant use of TR and VR tools may offer many opportunities to monitor and improve the health status of patients with SABI. TR may allow for continuity of service and care through the entire rehabilitation cycle, including assessment, intervention, consultation, and education, affording early reintegration and positively enhancing the QoL [30].

Among the TR systems, the Virtual Reality Rehabilitation System (VRRS) HomeKit device (Khymeia) enables the patient to carry out the training program at home, completely supervised by the therapist through a remote workstation [31]. The TR tool is equipped with VR that provides the patient with the ability to perform repetitive and goal-oriented tasks, with exercises with gradually increasing difficulty, promoting the motivation and overall functioning of the patient [32]. Moreover, to also focus on caregivers’ needs, evaluating their level of distress, emotional burnout, and psychological well-being is necessary. In fact, in our opinion, their role as cotherapists and their direct interaction during training are essential elements of a good neurorehabilitation process. The presence of a collaborative caregiver may indeed help obtaining better outcomes for the patient.

The main purpose of this multicenter randomized controlled trial (RCT) was to test the effectiveness of advanced training provided using the VRRS HomeKit device in improving functional outcomes in patients with SABI. Moreover, we sought to investigate the effects of TR on the psychological well-being of caregivers of patients with SABI.

## Methods

### Study Population

**Figure 1:**
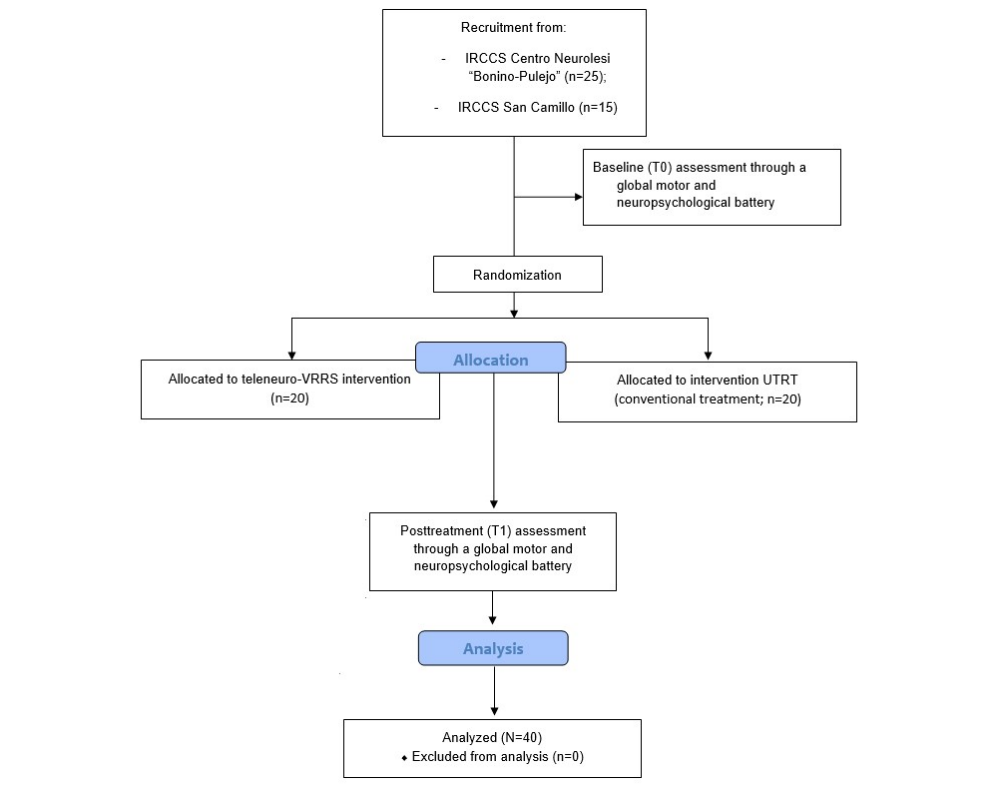
Study flow diagram adapted from Consolidated Standards of Reporting Trials (CONSORT) 2010. IRCCS: Istituto di Ricovero e Cura a Carattere Scientifico; UTRT: usual territorial rehabilitative treatment;VRRS: virtual reality rehabilitation system.

This study enrolled 40 subjects with SABI (n=23, 58%, males and n=17, 42%, females) and their 40 caregivers (n=14, 35%, males and n=26, 65%, females) who visited the Intensive Neurorehabilitation Care Unit of the Istituto di Ricovero e Cura a Carattere Scientifico (IRCCS) Centro Neurolesi “Bonino-Pulejo” (Messina, Italy) and the IRCCS San Camillo (Venice, Italy) from October 2018 to August 2022 (see Figure 1 and Multimedia Appendix 1).

### Ethical Considerations

The study was conducted according to the ethical policies and procedures approved by the local ethics committee Centro Neurolesi Bonino Pulejo (IRCCS-ME 08/2018), and it is part of a registered RCT (NCT03709875). All patients’ legal guardians provided written informed consent for study participation and data publication.

### Procedures

Patients’ inclusion criteria were (1) a diagnosis of SABI (vascular or traumatic etiology) in the subacute/chronic phase (ie, at least 6 months after the event), (2) age range between 18 and 75 years, (3) the presence of a stable internet connection, and (4) the presence of a caregiver able to use simple digital devices.

Patients’ exclusion criteria were (1) severe cognitive and behavioral impairments, (2) cardiorespiratory instability or other medical illness potentially interfering with treatment, (3) severe limb spasticity (Modified Ashworth Scale [MAS] score>3), and (4) a high risk of spontaneous fracture.

Patients were randomly assigned to 1 of 2 groups using a web-based application for block randomization. We used the block randomization method (block size=4) to ensure balance in the sample size across groups over time. Clinical evaluations were performed by therapists different from those who administered the treatments.

In both groups, each treatment (cognitive or motor or both as per patients’ functional status) lasted about 1 hour/day, 5 days/week, for 12 weeks.

The teleneuro-VRRS group received advanced motor and cognitive training at home with the VRRS HomeKit device, with the caregivers acting as cotherapists, whereas the control group was administered usual territorial rehabilitative treatment (UTRT, physical) and cognitive training at home. Before discharge, patients and caregivers were instructed on the use of the VRRS HomeKit device, and the training section was simulated within the hospital setting to find out potential problems and assess usability [33]. Both patients and caregivers had 3 meetings with the telemedicine operators to be provided with the basic information for the correct use of the tool. Next, the patients underwent 6 simulation training sessions (3 times/week for 2 weeks, each session lasting about 1 hour). The TR simulation was carried out using the Tele-Cockpit workstation and the same VRRS HomeKit device the patients would have used at home. The instruction training session was scheduled at another time if the caregiver or patient felt insecure about using the system.

### Outcome Measures

Each patient received a complete motor and neuropsychological evaluation before and immediately after the rehabilitation treatment (ie, at T0 and T1, respectively), administered by skilled physiotherapists, psychologists, or psychiatric therapists of both centers (IRCCS Centro Neurolesi and IRCCS San Camillo). The assessors were blind to the patients’ treatment. The motor evaluation included the Barthel Index (BI) [34] to assess the global functional status, the Tinetti Scale (TS) [35] to evaluate balance and gait recovery, and the MAS [36] to evaluate spasticity in upper limbs (shoulder, elbow, wrist) and lower limbs (hip, knee, ankle). The neuropsychological assessment included the Beck Depression Inventory II (BDI-II) to evaluate depression symptoms [37], the Montreal Cognitive Assessment (MoCA) to assess the global cognitive status [38], the Short Form Health Survey 36 (SF-36) [39] to measure the QoL, the Psychological General Well-Being Index (PGWBI) [40] for behavioral and mood problems, and the Frontal Assessment Battery (FAB) [41] to evaluate frontal abilities. In addition, the Caregiver Burden Inventory (CBI) [42] was administered to each caregiver to evaluate the emotional burden status (see Table 1).

**Table 1:** Neuropsychological and motor assessments used with patients with SABI^a^ and their caregivers.

Test/scale	Domains	Administered to	Description
BI^b^ [34]	Global functional status	Patients	The BI is an ordinal scale used to measure performance in ADL^c^. In total, 10 variables describing ADL and mobility are scored, with higher scores reflecting a greater ability to function independently following hospital discharge.
TS^d^ [35]	Balance and gait	Patients	The test includes 2 short sections, one examining static balance abilities in a chair and then standing and the other gait. The Tinetti test has a gait score and a balance score. It uses a 3-point ordinal scale of 0, 1, and 2. Gait is scored over 12, and balance is scored over 16, totaling 28.
MAS^e^ [36]	Spasticity	Patients	The MAS is a muscle tone assessment scale used to assess the resistance experienced during passive range of motion for upper and lower limbs, which does not require any instrumentation and is quick to perform.
BDI-II^f^ [37]	Depression symptoms	Patients	The BDI-II is a widely used measure; it is a 21-item self-report inventory measuring the severity of depression symptoms (cut-off>10).
MoCa^g^ [38]	Global functioning cognition	Patients	MoCA is a cognitive screening test questionnaire used in the detection of mild/severe cognitive impairment (cut-off<26). MoCA assesses multiple cognitive domains, including attention, concentration, executive functions, memory, language, visuospatial skills, abstraction, calculation, and orientation. It is a paper-and-pencil tool that requires approximately 10 minutes to administer and is scored out of 30 points.
SF-36^h^ [39]	Impact of a disease (QoL^i^)	Patients	The SF-36 is a generic, multidimensional tool consisting of 36 questions that can be divided into 8 subscales.
PGWBI^j^ [40]	General well-being	Patients	The PGWBI consists of 22 self-administered items rated on a 6-point scale, which assess the psychological and general well-being of respondents in 6 health-related QoL domains: anxiety, depression, positive well-being, self-control, general health, and vitality.
FAB^k^ [41]	Executive functions	Patients	The FAB is a short clinical battery of 6 neuropsychological subtests designed to assess frontal lobe function. The 6 FAB tasks evaluate cognitive and behavioral domains that are thought to be under the control of the frontal lobe: conceptualization and abstract reasoning, lexical verbal fluency and mental flexibility, motor programming and executive control of action, self-regulation and resistance to interference, perseveration, and inhibitory control behavior and environmental autonomy (cut-off<12).
CBI^l^ [42]	Distress symptoms	Caregivers	The CBI is a 24-item Likert-format scale (score 0-4) that measures 5 dimensions of caregiver burden: time dependence, developmental, physical, social, and emotional.

^a^SABI: severe acquired brain injury.

^b^BI: Barthel Index.

^c^ADL: activities of daily living.

^d^TS: Tinetti Scale.

^e^MAS: Modified Ashworth Scale.

^f^BDI-II: Beck Depression Inventory II.

^g^MoCa: Montreal Cognitive Assessment.

^h^SF-36: Short Form Health Survey 36.

^i^QoL: quality of life.

^j^PGWBI: Psychological General Well-Being Index.

^k^FAB: Frontal Assessment Battery.

^l^CBI: Caregiver Burden Inventory.

### Usual Territorial Rehabilitative Treatment

UTRT consisted of both cognitive and motor programs in a face-to-face setting. During the study period, patients were followed at home by a physiotherapist (2-3 times/week) or a speech therapist (2-3 times/week) according to their individualized rehabilitation program. Treatments for motor limbs activity focused on functional active-assisted and active exercises (eg, reaching or gait movements), in addition to treatments to improve balance and posture on functional exercises (eg, standing up or sitting down, changing direction and speed, and walking upstairs, when possible) and treatments to ameliorate the execution of common activities of daily living (ADL), such as feeding, washing, and toileting. Conventional paper-and-pencil training was used to improve multiple cognitive functions through specific rehabilitative programs for (1) attention processes and concentration, (2) verbal and visuospatial memory abilities, and (3) executive functions. These programs consisted of a series of exercises using standard rehabilitative materials (eg, images, colors, barrage tasks) and a face-to-face approach with the cognitive therapist in a quiet environment, without disturbing noises or distractions (see Tables 2-5).

**Table 2:** SABI^a^ cognitive neurorehabilitation program with motor exercises: VRRS^b^ HomeKit vs conventional UTRT^c^.

Exercise	UTRT	VRRS HomeKit
Eye-hand coordination	Eye-hand coordination exercises include goal-directed movement, with the support of the therapist, to collect objects of various shapes, colors, and sizes. The main goal is to increase active movements in the upper limbs, and manipulative exercises are proposed through active-passive mobilization, reaching, and pointing activities.	Manual eye coordination exercises include the visual achievement of colored targets (eg, balls, machines, asteroids) to select or collect them by moving the upper limbs using a K-wand sensor. The therapist may subsequently increase the rate of displacement of the moving targets, making the exercise more difficult. The main goal is to increase active movements in the upper limbs, and manipulative exercises are proposed with the K-wand sensor module, which includes a cylinder-like sensor that allows the patient unable to use the touch system to carry out activities of gripping, selecting, pointing, and reaching.
Trunk control	Trunk control exercises include sit-to-stand activities to improve postural changes from a sitting to a standing position, training for postural changing, standing still, and weight shifting without audio and video stimulation.	Trunk control exercises include both sitting activities with laterolateral and anteroposterior movements of the trunk and standing activities with the support of the caregiver. The patient, using sensors on the trunk, has to collect colored targets by moving the trunk and controlling the shift of the body weight to achieve improved control of reactive stepping (eg, faster reaction time).
Bimanual coordination	Bimanual coordination activities are carried out through exercises working on the skills of the upper limbs and hands. In a sitting position, different types of balls, both in shape and in size, and other objects are used to exercise the fingers and fingertips. The exercises can be simplified by modulating the distance of the chosen ball or object.	Exercises for carrying out bimanual coordination activities are based on trying to catch targets (eg, machines, balls, water drops via a virtual umbrella) in motion and in all directions, associated with audio and video stimulation. The therapist can choose the number of targets for the patient to select and the time within which the exercise should be performed.

^a^SABI: severe acquired brain injury.

^b^VRRS: virtual reality rehabilitation system.

^c^UTRT: usual territorial rehabilitative treatment.

**Table 3:** SABI^a^ cognitive neurorehabilitation program with cognitive exercises: VRRS^b^ HomeKit vs conventional UTRT^c^.

Exercise	UTRT	VRRS HomeKit
Selective attention processes	This includes indicating and touching directly with the hand the selected/standard target stimuli in relation to specific characteristics presented (eg, colors, images, animals, functions), neglecting distractions, which consist of other pictures different in the number and complexity of criteria. A cognitive therapist provides verbal commands to the patient, which combines the different selective images. The patient touches the standard target stimuli presented in a specific time according to the therapist’s verbal command.	This includes selecting and immediately recalling the same feedback (audio and video) of various elements (eg, colors, musical strings, geometric forms or not, animals) observed in the virtual environment. The patient touches the virtual target element in a specific time; this action causes a visual change with specific audio feedback (positive reinforcement), using the VVRS interaction between the cognitive therapist and the patient. Otherwise, the element disappears (negative reinforcement).
Sustained attention processes	To stimulate sustained attention processes, the patient observes 3-5 target stimuli for a variable and progressive amount of time (10-15 minutes), with an attentional focus on traditional tasks.	The patient observes 3-5 target stimuli for a variable and progressive amount of time (10-15 minutes), with an attentional focus on virtual tasks. These elements remain visible to the observer for a variable amount of time established by the interaction between the virtual system, the therapist, and the patient.

^a^SABI: severe acquired brain injury.

^b^VRRS: virtual reality rehabilitation system.

^c^UTRT: usual territorial rehabilitative treatment.

**Table 4:** SABI^a^ cognitive neurorehabilitation program for memory abilities: VRRS^b^ HomeKit vs conventional UTRT^c^.

Exercise	UTRT	VRRS HomeKit
Verbal	Memory-training tasks are carried out with paper and pencil in a face-to-face rehabilitative setting, with an interaction only between therapist and patient. These tasks include recalling the locations of a series of items on a specific rehabilitative table (in a cognitive room), recalling digits or letters in either the order presented or reverse order, or recalling specifically where a particular number, name, or digit was in a sequence.	In these memory-training tasks, the patient is asked to observe at first particular elements and then (in the immediate and recall time) to remember those (eg, eggs, seasons, colors, balls, numbers, environments, animals, geometric forms or not, fruits, jobs) with a dynamic interaction in a semi-immersive virtual environment (using sprites tasks). The patient must remember the place (the position; visuospatial memory) and name (verbal information) of the element observed.
Visuospatial	The goal is to process visual stimuli, to comprehend spatial relationships between objects and to visualize different scenarios or images, to record and recover information needed to plan a course to a location, and to recall the virtual location/position of an object or the occurrence of an event. The patient must remember the place (the position; visuospatial memory) and name (verbal information) of the element observed.	The goal is to process visual stimuli to comprehend spatial relationships between virtual objects and to visualize different virtual scenarios or computer-based images, to record and recover information needed to plan a course to a location, and to recall the virtual location/position of an object or the occurrence of an event.

^a^SABI: severe acquired brain injury.

^b^VRRS: virtual reality rehabilitation system.

^c^UTRT: usual territorial rehabilitative treatment.

**Table 5:** SABI^a^ cognitive neurorehabilitation program for executive functions: VRRS^b^ HomeKit vs conventional UTRT^c^.

Exercise	UTRT	VRRS HomeKit
Verbal fluency	This involves working on categorization (semantic and phonemic) without the use of virtual tools. Some movements finalized to direct touch and to move specific objects (eg, pencils, pens) in different directions (eg, left, right) are programmed.	This involves working on categorization (semantic and phonemic), planning, association, and analogical reasoning using a computer-based approach. Some movements finalized to virtually touch, move, or manipulate specific objects (eg, balls, flowers, butterflies) in different directions are programmed.
Reasoning	This involves planning, association, and analogical reasoning without the use of virtual tools according to the therapist’s indication in a specific space (eg, rehabilitative table) or realizing specific associations (eg, letter-color), using a pen-and-pencil approach, organized in a face-to-face rehabilitative session.	This involves planning, association, and analogical reasoning using a computer-based approach or realizing specific associations (eg, number-color) with a dynamic interaction in a virtual environment. When the patient touches virtual objects, they obtain video and audio feedback (using sprites tasks). In particular, the patient realizes ideomotor sequences (from simple to complex series of actions) after the verbal command of the therapist.

^a^SABI: severe acquired brain injury.

^b^VRRS: virtual reality rehabilitation system.

^c^UTRT: usual territorial rehabilitative treatment.

### Teleneurotraining Using the VRRS HomeKit

**Figure 2:**
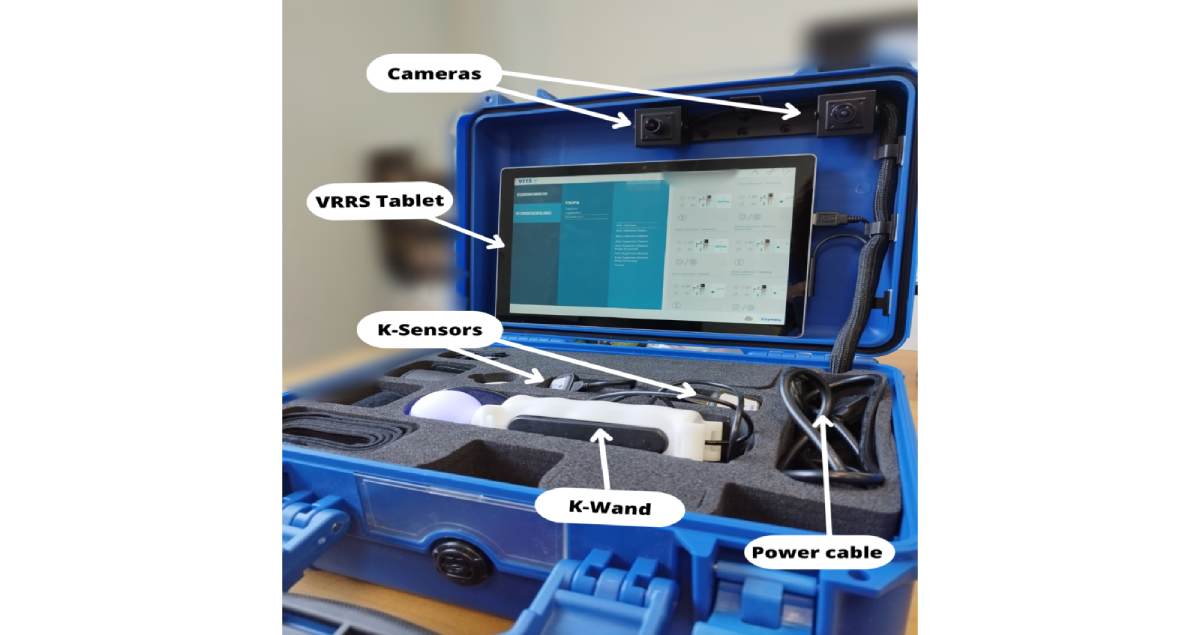
The VRRS HomeKit used to train patients with SABI in TR modality. SABI: severe acquired brain injury; TR: telerehabilitation; VRRS: virtual reality rehabilitation system.

The experimental training was conducted using the VRRS HomeKit (Figure 2). The device is a tablet placed in a carrying case, with sensors, such as a K-wand and K-sensors, that allows conducting a complete training program at home, including motor, cognitive, and speech therapy modules. During each training session, the patient is supported by the therapist (neuropsychologist or physiotherapist) through the teleworkstation (ie, Tele-Cockpit) and by the caregiver in the role of a cotherapist to assist the therapist. Tele-Cockpit is an innovative technological workstation equipped with a proprietary integrated videoconferencing system for the management of remote and home devices, such as the VRRS HomeKit. The system includes teletraining, telemonitoring, teleconsultation, and streaming of diagnostic imaging. Tele-Cockpit allows the therapist to take control of the remote device and simultaneously see what the patient is doing on the VRRS HomeKit, fully interacting with them in real time.

Teleneuro-VRRS sessions included the stimulation of specific cognitive domains, such as attention, verbal and visuospatial memory, and executive functions, as well as motor function training, using the advanced system, the VRRS HomeKit. The exercises were presented with increasing difficulty and implemented according to the patient’s response to the treatment (ie, when 9 of 10 answers were correct) and the number of errors (errors<1). Additionally, the therapists could also select the duration of each virtual exercise to better personalize both motor and cognitive tasks according to patients’ abilities, attention levels, and needs.

Each session lasted about 1 hour (the same as the URTRs), although some extra time (less than 15 minutes) was also necessary for technology issues, including low bandwidth, content glitches, and comfort with the system linked to the distance of the user or the height at which the device is placed in the home setting.

The VRRS HomeKit allows nonimmersive virtual exercises where the patient interacts with 2D scenarios and objects through the touch screen or through particular sensors: a K-wand equipped with light recognition technology used for movement tracking and orientation, which is handled by the patient during catching and reaching virtual exercises for the upper limbs, and a K-sensor consisting of a set of sensors placed on wearable strips of different sizes, which is used to carry out full-body motor teletraining activities. This means that although nonimmersive VR is in both cases represented on the same flat screen, the upper and lower limbs can move in the 3 dimensions of the space while interacting with the virtual environment. The entire cost of the system (Tele-Cockpit plus HomeKit) was €36,000 (USD 39,508): each home kit cost €8000 (USD 8780), and we bought 4 of them to ensure proper study training); see Tables 2-5.

### Statistical Analysis

Continuous variables were expressed as mean and SD values (ie, age and time since injury) or first-third quartiles (ie, psychometric and outcome measures), as appropriate. Categorical variables (ie, education and etiology) were expressed as frequencies and percentages. The normal distribution of the sample was investigated through the Shapiro-Wilk test, whereas the Levene test was performed to assess the equality of variances among times for each variable. According to the reduced sample dimension and nonnormality of all variables, we chose a nonparametric analysis. Thus, where appropriate, outcome measures and psychometric variables were analyzed using the 1- or 2-tailed Wilcoxon signed-rank test to detect changes between baseline (T0) and posttreatment (T1) periods within the same group (intragroup analysis); in contrast, the comparison of continuous variables and outcome measures between the 2 groups (intergroup analysis), at both T0 and T1, was performed considering the mean of the Mann-Whitney U test. Linear correlation between variables was calculated with the nonparametric Spearman rank correlation coefficient. In addition, we calculated the effect sizes (ESs) for each psychometric and outcome measure through the Cohen d test between 2 groups to describe whether the treatment effects achieved had a sufficient magnitude. Statistical significance was set at a bilateral α level of .05. Each analysis was performed on the open source software R 4.1.3 (R Foundation for Statistical Computing) for Windows (Microsoft Corporation) [43].

In this study, we did not perform an intention-to-treat analysis because heterogeneity might have been introduced if noncompliant subjects, dropouts, and compliant subjects were mixed in the final analysis, with potential concerns also around the real/potential efficacy of the treatments [44].

## Results

### Participant Characteristics

The mean age of the 40 patients was 48.12 (SD 16.84) years. Of them, 12 (23%) had a traumatic etiology and 28 (77%) a vascular etiology, with a time since injury from 6 to 18 months (mean 11.43, SD 3.45). A more detailed description of both patients’ and caregivers’ demographic characteristics is reported in Tables 6 and 7, respectively.

All subjects completed the training, and no adverse event was reported. We found no significant differences between the 2 groups (including patients and caregivers) in age, gender, and education (Tables 6 and 7). At baseline (T0), our analysis showed no significant differences between the outcome scores of the 2 groups (Table 8).

**Figure 3:**
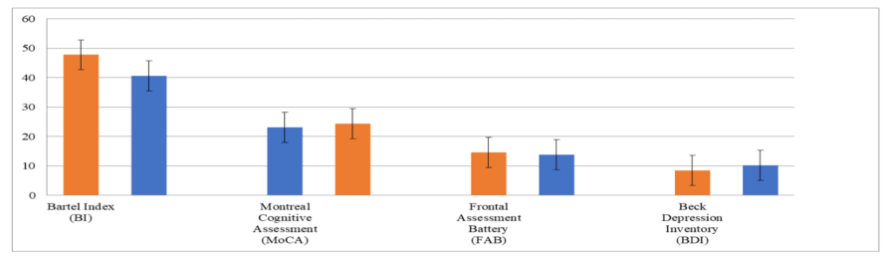
The histogram compares the main functional and cognitive psychometric data obtained posttreatment (T1) between the teleneuro-VRRS group (orange) and the control group (conventional training, blue). VRRS: virtual reality rehabilitation system.

**Figure 4:**
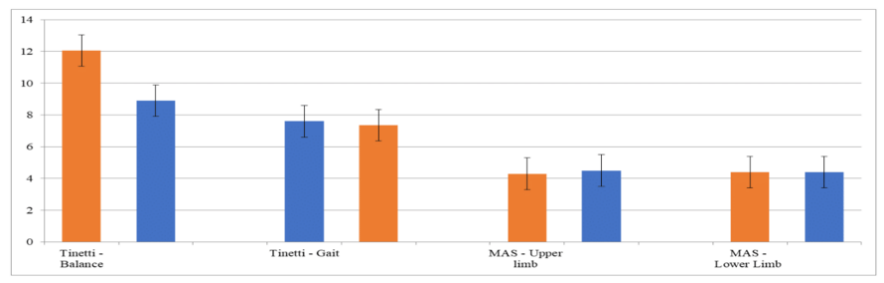
The histogram compares the psychometric data (TS and MAS) obtained posttreatment (T1) between the teleneuro-VRRS group (orange) and the control group (conventional training, blue). MAS: Modified Ashworth Scale; TS: Tinetti Scale; VRRS: virtual reality rehabilitation system.

In the teleneuro-VRRS intragroup analysis (Table 9), comparing baseline (T0) and posttreatment (T1) periods, we found statistical differences in the BI (*P*<.001), FAB (*P*<.001), BDI-II (*P*<.001), and MoCA (*P*<.001); the TS for balance (*P*<.03) and gait (*P*<.02); and the CBI (*P*<.004). See also Figures 3 and 4.

Moreover, by analyzing the SF-36 subtests, we found statistically significant differences between baseline (T0) and posttreatment (T1) periods in physical functioning (*P*<.01), physical role functioning (*P*<.001), emotional role functioning (*P*<.008), and general health perception (*P*<.001). The PGWBI results were extremely significant in all subtests, as reported in Table 8.

In the baseline (T0) and posttreatment (T1) analysis of the control group, statistically significant differences emerged in the BI (*P*<.001) and the TS for gait (*P*<.03), as well as in MoCA (*P*<.001). In the SF-36, only the physical functioning (*P*<.004), bodily pain (*P*<.01), and general health perception (*P*<.004) subtests showed statistical significance (Table 9). Moreover, the general health (*P*<.004) and vitality (*P*=.05) PGWBI subtests were statistically significant.

We calculated the usability score with the System Usability Scale (SUS; median=69.00, first quartile=65.00, third quartile=76.25), indicating high user satisfaction with the tool’s usability. Between-group analysis (Table 8) showed statistical differences in the anxiety (*P*<.02, ES=0.85) and self-control (*P*<.03, ES=0.40) subtests of the PGWBI and in the social role functioning (*P*<.02, ES=0.85) subtest of the SF-36. This was also confirmed by the quite medium and large ESs.

**Table 6:** Demographic and clinical characteristics of patients with SABI^a^ in teleneuro-VRRS^b^ and control groups.

Characteristics			All participants (N=40)		Control group (n=20)		Teleneuro-VRRS group (n=20)
Age (years), mean (SD); *P*=.058^c^			48.12 (16.84)		43.4 (15.78)		52.85 (16.90)
**Gender, n (%); *P*=.99^c^**							
	Male	23 (58)		11 (55)		12 (60)	
	Female	17 (42)		9 (45)		8 (40)	
**Education (years), n (%); *P*=.99^c^**							
	Elementary school	14 (35)		5 (25)		8 (40)	
	Middle school	18 (44)		13 (65)		6 (30)	
	High school	7 (18)		2 (10)		5 (25)	
	University	1 (3)		0		1 (5)	
**Etiology, n (%); *P*=.73^c^**							
	Vascular	28 (70)		15 (75)		13 (65)	
	Traumatic	12 (30)		5 (25)		7 (35)	
Time since injury, mean (SD); *P*=.89^c^			11.43 (3.45)		10.76 (3.68)		12 (3.22)

^a^SABI: severe acquired brain injury.

^b^VRRS: virtual reality rehabilitation system.

^c^*P* values at baseline (T0) between the 2 groups.

**Table 7:** Demographic and clinical characteristics of caregivers (N=40) of patients with SABI^a^ in teleneuro-VRRS^b^ and control groups.

Characteristics			All participants (N=40)		Control group (n=20)		Teleneuro-VRRS group (n=20)
Age (years), mean (SD); *P*=.96^c^			51.1 (12.77)		50.4 (14.33)		51.8 (11.33)
**Gender, n (%); *P*=.32^c^**							
	Male	14 (35)		11 (55)		5 (25)	
	Female	26 (65)		9 (45)		15 (75)	
**Education (years), n (%)^d^; *P*=.72^c^**							
	Elementary school	6 (15)		4 (20)		2 (10)	
	Middle school	19 (48)		7 (35)		12 (60)	
	High school	14 (35)		8 (40)		6 (30)	
	University	1 (3)		1 (5)		0	

^a^SABI: severe acquired brain injury.

^b^VRRS: virtual reality rehabilitation system.

^c^*P* values at baseline (T0) between the 2 groups.

^d^The percentages might add up to more than 100 because of rounding.

**Table 8:** Statistical comparison using the Mann-Whitney U test between teleneuro-training using the VRRS^a^ HomeKit and conventional UTRT^b^ for neuropsychological evaluation and their ES^c^.

Psychometric test/scale^d^ and domain		Baseline (T0) *P* value between 2 groups	Posttreatment (T1) *P* value in between-group analysis	ES
BI^e^ (global functional status)		.88	.35	0.26
**TS^f^ (gross motor functions)**				
	Balance	.31	.09	0.82
	Gait	.85	.91	0.08
**MAS^g^**				
	Spasticity, upper limb	.37	.96	0.05
	Spasticity, lower limb	.70	.81	0.11
MoCA^h^ (global cognitive index)		.40	.47	0.33
BDI-II^i^ (depression symptoms)		.08	.11	0.31
FAB^j^ (executive functions)		.34	.50	0.04
CBI^k^ (caregiver’s burden)		.64	.62	0.10
**SF-36^l^**				
	Physical functioning	.87	.96	0.10
	Limitation in physical role functioning	.40	.18	0.39
	Bodily pain	.70	.96	0.04
	Vitality	.77	.37	0.23
	General health perceptions	.91	.27	0.20
	Mental health	.59	.53	0.08
	Emotional role functioning	.12	.20	0.05
	Social role functioning	.69	.02^m^	0.85
**PGWBI^n^**				
	Positive well-being	.87	.18	0.38
	General health	.77	.16	0.48
	Depressed mood	.97	.05	0.61
	Vitality	.68	.13	0.43
	Self-control	.87	.03^m^	0.40
	Anxiety	.99	.02^m^	0.85

^a^VRRS: virtual reality rehabilitation system.

^b^UTRT: usual territorial rehabilitative treatment.

^c^ES: effect size.

^d^Specific motor and cognitive domains are reported in parentheses.

^e^BI: Barthel Index.

^f^TS: Tinetti Scale.

^g^MAS: Modified Ashworth Scale.

^h^MoCa: Montreal Cognitive Assessment.

^i^BDI-II: Beck Depression Inventory II.

^j^FAB: Frontal Assessment Battery.

^k^CBI: Caregiver Burden Inventory.

^l^SF-36: Short Form Health Survey 36.

^m^The *P* value is significant at a significance level of .05 (2-tailed).

^n^PGWBI: Psychological General Well-Being Index.

**Table 9:** First-third quartiles, obtained through the Wilcoxon signed-rank test, of psychometric tests administered to patients with SABI^a^ and their caregivers, between teleneuro-training using the VRRS^b^ HomeKit (teleneuro-VRRS) and conventional UTRT^c^ (control), and statistical intragroup analysis at baseline (T0) and posttreatment (T1).

Psychometric test/scale^d^ and domain		Range (first-third IQR)				T0-T1 *P* value (intragroup analysis)	
		Teleneuro-VRRS	Control		Teleneuro-VRRS		Control
BI^e^ (global functional status)		35.00-58.75	13.75-66.75		<.001^f^		<.001^f^
**TS^g^ (gross motor functions)**							
	Balance	11.00-15.00		5.75-11.00	<.03^f^		.90
	Gait	6.00-10.00		5.25-10.00	<.02^f^		.03^f^
**MAS^h^**							
	Spasticity, upper limb	3.00-6.00		4.50-6.00	.90		.90
	Spasticity, lower limb	3.00-6.00		4.50-6.00	.70		.70
MoCA^i^ (global cognitive index)		20.00-26.00	21.75-26.25		<.001^f^		<.001^f^
BDI-II^j^ (depression symptoms)		7.00-12.25	9.00-12.25		<.001^f^		.14
FAB^k^ (executive functions)		14.00-14.00	13.44-16.30		<.001^f^		.58
CBI^l^ (caregiver’s burden)		27.00-49.00	13.75-58.50		<.004^f^		.07
**SF-36^m^**							
	Physical functioning	0.00-13.75		0.00-43.25	<.01^f^		<.004^f^
	Limitation in physical role functioning	0.00		0.00-30.00	<.001^f^		<.05
	Bodily pain	37.50-88.00		75.00-90.00	.50		<.01^f^
	Vitality	25.00-45.00		40.00-60.00	.18		.74
	General health perceptions	25.00-45.00		35.00-56.25	<.001^f^		<.004^f^
	Mental health	47.25-65.25		47.25-61.25	.20		.10
	Emotional role functioning	24.75-100.00		35.00-86.25	<.008^f^		.99
	Social role functioning	25.00-65.25		30.00-72.50	.11		.62
**PGWBI^n^**							
	Positive well-being	35.00-58.75		24.75-45.50	<.002^f^		.08
	General health	43.75-65.00		38.75-56.25	<.004^f^		.004^f^
	Depressed mood	69.00-90.75		59.50-72.25	<.005^f^		.40
	Vitality	45.00-57.50		41.75-55.00	<.001^f^		<.05^f^
	Self-control	57.00-70.00		49.50-61.00	<.001^f^		.21
	Anxiety	68.75-85.00		57.25-72.00	<.004^f^		.91

^a^SABI: severe acquired brain injury.

^b^VRRS: virtual reality rehabilitation system.

^c^UTRT: usual territorial rehabilitative treatment.

^d^Specific motor and cognitive domains are reported in parentheses.

^e^BI: Barthel Index.

^f^The *P* value is significant at a significance level of .05 (2-tailed).

^g^TS: Tinetti Scale.

^h^MAS: Modified Ashworth Scale.

^i^MoCa: Montreal Cognitive Assessment.

^j^BDI-II: Beck Depression Inventory II.

^k^FAB: Frontal Assessment Battery.

^l^CBI: Caregiver Burden Inventory.

^m^SF-36: Short Form Health Survey 36.

^n^PGWBI: Psychological General Well-Being Index.

## Discussion

### Principal Findings

This multicenter RCT sought to investigate the efficacy of a TR nonimmersive VR system on the motor performance and cognitive abilities of patients with SABI compared to a control group undergoing conventional training (URTRs). Our results indicated that both teleneuro-VRRS and control groups improved in global functional, cognitive, and general health status. However, we found that only the teleneuro-VRRS group improved in motor and executive functions, with a significant reduction in anxiety and depression symptoms. These findings confirmed the results of a previous study carried out on patients with stroke [23].

The current literature mostly focuses on the usability and feasibility of TR systems [33], whereas their efficacy has been less investigated, especially in TBI. Indeed, to the best of our knowledge, this is the first study to assess the effects of a teleneuro-VRRS on motor and cognitive outcomes using a specific TR program in patients with SABI. In fact, other authors have used scheduled telephone interviews or internet-based approaches [24] for educational interventions and exercise maintenance without a rehabilitation program. Nonetheless, patients with SABI perceived our approach as optimal and satisfying, because it may guarantee the continuity of care. In this aspect, the TR modality allows constant and intensive treatment, thanks to its easy use and the possibility of asynchronous sessions, whereas UTRT depends exclusively on the presence of therapists and is usually limited to about 3 times/week. In line with our data, Solana et al [23] showed how TR using a computerized platform improves cognitive functioning, with regard to attention, memory, and executive functions, in people with TBI. The teletraining also increased the efficiency of the rehabilitation process, facilitating access to treatment and reducing the associated costs. Moreover, a VR system can play a crucial role in the patient’s functional recovery, since it encourages more active participation and allows for longer training sessions. In our teleneuro-VRRS sample, the VRRS increased patients’ motivation and enjoyment, both important factors for successful rehabilitative treatment [26,27].

In a VR environment, exercises favor the development of the knowledge of (1) results of movements and (2) quality of movements (performance) [28,29,45,46]. This way, VR can stimulate the central nervous system, which receives increased feedback signals (augmented feedback), inducing profound changes in neural plasticity, responsible for motor and cognitive function recovery [30,31,47-50]. In this context, better results may be achieved by coupling different tools. Indeed, the emerging use of neuromodulation demonstrates a positive effect by promoting neuroplasticity and functional recovery following a brain injury [51-53]. Eilam-Stock et al [54] studied the promising effects of a combined approach using a TR system plus neuromodulation (ie, transcranial direct current stimulation [tDCS]) in a patient with brain injury. The authors reported improvements in specific cognitive domains, such as attention and working memory, processing speed, and semantic fluency. This may be consistent with the site of stimulation (ie, the left dorsolateral prefrontal cortex) that is involved directly and indirectly in these cognitive processes. Further studies using the combined approach are needed to demonstrate whether and to which extent this could be effective in patients with SABI.

Another important finding of our study is the more evident reduction in the burden of distress in the caregivers of patients in the teleneuro-VRRS group compared to the control group. This is probably why caregivers were involved during the TR sessions, acting as cotherapists, but received, at the same time, support from a multidisciplinary rehabilitation team for their social, physical, and psychological needs. In fact, remote interventions can be effective in reducing psychological distress, thanks to the active participation in the care and decision-making processes, also avoiding travel difficulties related to access of appropriate health care providers [55-57].

Lastly, we investigated the usability of the TR system by means of the SUS score, demonstrating that subjects perceived the system as efficient, more satisfying, and easier to use, in line with the literature [58-61]. To overcome the challenges in the use of technologies for some patients with SABI, training both patients and caregivers when they are at the rehabilitation center could be of help [13].

### Strengths, Limitations, and Future Perspectives

The strengths of the study are linked to the development and implementation of complete and dedicated motor and cognitive TR training using a VR system, whereas most of the previous studies have mainly focused on feasibility/usability. In addition, our VR system was available in the asynchronous modality to enable patients to undergo repetitive and intensive training, even without the direct supervision of the therapist. In addition, the caregivers acted as cotherapists during the rehabilitation session, reducing their burden and efforts and helping improve patient outcomes. Indeed, the growing literature demonstrates the importance of family members in the rehabilitation pathway and the patient’s reintegration [62].

The main limitations of the study are the relatively small sample size that prevents us from generalizing our promising results to the entire SABI population, in addition to the lack of follow-up. Nonetheless, it was difficult to enroll a larger and homogenous SABI sample, also considering the organizational problems related to the COVID-19 pandemic. Another concern may be that we included both vascular and traumatic SABI, which are known to have different recovery patterns and functional outcomes. However, the aim of the study was to investigate the feasibility and potential efficacy of TR in this patient population to pave the way for the continuity of care.

The lack of “human touch” and face-to-face interaction, which is a well-known special tool for nonverbal communication with patients [63], is the main pitfall of the use of TR, in addition to the scarce usability by older patients. We investigated the latter issue in a previous work, with positive results [13]. In addition, the reduced/lack of internet availability in rural settings and vision difficulties due to the small screen size of the tablet, often associated with poor sound quality, are other important concerns to acknowledge when dealing with TR.

Considering that TR is a growing and potentially efficacious field, it should be analyzed in terms of cost and effectiveness [64]. In fact, this analysis should include the cost of technologies (ie, the VR system) and equipment, the internet, and specific maintenance services. In this aspect, the primary savings due to the use of TR for patients with neurological issues are related to the absence of in-person therapist costs and mileage reimbursement [64]. However, the costs of face-to-face rehabilitation include the therapist’s service and travel expenses for both the therapist (ie, domiciliary therapy) and the caregiver (ie, ambulatory care) in terms of money, time spent, and effort [65]. Nevertheless, a combination of both approaches (TR and UTRT) will meet the needs of those patients who cannot travel or have a higher economic burden. Future studies must analyze the economic issue, comparing TR and UTRT costs for patients with neurological issues, with regard to those with SABI. Lastly, future research in the TR field should also measure the group-by-time interaction effect, analyzing the extent to which the difference between groups varies at different occasions, through specific statistical tests, such as repeated measures ANOVA [66].

### Conclusion

Our study demonstrates that TR using nonimmersive VR may improve functional outcomes in patients with SABI. Indeed, TR could be considered a suitable alternative to traditional rehabilitation in this patient population to guarantee the continuity of care after discharge, especially in remote or underserved areas. Larger-sample multicenter studies are needed to further investigate the effects of TR when using more patient-tailored motor and cognitive training programs, also considering the cost-benefit analysis.

## Appendix

Multimedia Appendix 1 CONSORT E-HEALTH checklist (V 1.6.1).

## Abbreviations

ADL: activities of daily living
BDI-II: Beck Depression Inventory II
BI: Barthel Index
CBI: Caregiver Burden Inventory
ES: effect size
FAB: Frontal Assessment Battery
IRCCS: Istituto di Ricovero e Cura a Carattere Scientifico
MAS: Modified Ashworth Scale
MoCa: Montreal Cognitive Assessment
PGWBI: Psychological General Well-Being Index
QoL: quality of life
RCT: randomized controlled trial
SABI: severe acquired brain injury
SF-36: Short Form Health Survey 36
SUS: System Usability Scale
TBI: traumatic brain injury
TR: telerehabilitation
TS: Tinetti Scale
UTRT: usual territorial rehabilitative treatment
VR: virtual reality
VRRS: virtual reality rehabilitation system

## Data Availability

Data are available upon request from the corresponding author.
